# Do patients know that physicians should be confidential? study on patients’ awareness of privacy and confidentiality

**Published:** 2018-02-13

**Authors:** Mohammad Mohammadi, Bagher Larijani, Seyed Hassan Emami Razavi, Akbar Fotouhi, Ahmad Ghaderi, Seyed Javad Madani, Mohammad Naser Shafiee

**Affiliations:** 1 *PhD Candidate in Medical Ethics, Medical Ethics and History of Medicine Research Center, Tehran University of Medical Sciences, Tehran, Iran; School of Medicine, Department of Medical Ethics, Tehran, University of Medical Sciences, Tehran, Iran; Faculty of Medicine, Department of Medical Ethics, Mashhad University of Medical Sciences, Mashhad, Iran. *; 2 *Professor, Endocrinology and Metabolism Research Center, Endocrinology and Metabolism Research Institute, Tehran University of Medical Sciences, Tehran, Iran. *; 3 *Associate Professor, Brain and Spinal Cord Injury Research Center, Neuroscience Institute, Tehran University of Medical Sciences, Tehran, Iran.*; 4 *Professor, Department of Epidemiology and Biostatistics, School of Public Health, Tehran University of Medical Sciences, Tehran, Iran. *; 5 *PhD Candidate in Medical Ethics, Medical Ethics and History of Medicine Research Center, Tehran University of Medical Sciences, Tehran, Iran; School of Medicine, Department of Medical Ethics, Tehran, University of Medical Sciences, Tehran, Iran. *; 6 *Assistant Professor, Department of English Language, Faculty of Medicine, Mashhad University of Medical Sciences, Mashhad, Iran.*

**Keywords:** *Privacy*, *Confidentiality*, *Awareness*, *Medical ethics*

## Abstract

Privacy and confidentiality are among the inalienable rights of every human being that contribute to preservation of a sense of reverence and dignity. The present study was conducted to examine patients’ awareness of their entitlement to these important rights.

This cross-sectional study was conducted on 200 patients in Tehran, Iran during the year 2010. Collected data included patients’ demographics (age, gender, marital status, place of residence, and educational level), type of hospital ward, frequency of hospitalization, duration of hospital stay, and patients’ awareness of privacy and confidentiality. Two trained interviewers gathered the data using a self-made questionnaire, which was specifically designed to assess patients’ awareness of privacy and confidentiality. Validity and reliability of the questionnaire were determined using content validity and Cronbach's Coefficient Alpha (a = 0.7), respectively. To analyze data, patients were assigned to three categories of poor (0 ≤ scores ≤ 3), moderate (4 ≤ scores ≤ 7) and good (8 ≤ scores ≤ 10) levels of awareness. Statistical analysis was performed by SPSS software version 21.

The results showed that 21% of the patients had poor, 72% moderate, and 7% good awareness of privacy and confidentiality, with a mean of 4.61 ± 1.63. In this study, 153 patients (76.5%) provided a correct definition of privacy, and 161 patients (80.5%) were aware of instances of privacy violation. In addition, a good level of awareness was found in 77 patients (38.5%) in terms of physician confidentiality, and in 158 patients (81.4%) regarding confidentiality of examination results and medical consultations. Our study results highlight the necessity to inform patients about the ethical and legal issues related to privacy and confidentiality, before or during admission.

## Introduction

Privacy and confidentiality are among the main features of ethical medical practice and are of great significance in all communities (1). Confidentiality is regarded by the UK General Medical Council and many other organizations as an ethical obligation (1). Chapter 4 of the Iranian Patients’ Rights Charter states that every individual has the right to privacy and confidentiality (2). Privacy refers to an individual’s control over how much, when, and under what circumstances they may share details of their physical, behavioral, or intellectual life with others, and their right to restrict other people’s access to their personal information (3). 

Thus, confidentiality means that providing information to another person will create a commitment on his or her part not to reveal it to anyone else. Accordingly, both that individual and the community will expect that information trusted to a health professional in a clinical context not be revealed to third parties. Hospitalized patients have limitations that may jeopardize their privacy and thus result in serious consequences (4). Moreover, confidentiality commitment provides the basis for trust in therapeutic communication.

Respecting the confidentiality of patient information is a major principle in ethics and effective medical activities. Confidentiality guarantees patients’ privacy (5). From the perspective of ethics, competent patients must be in full control of their personal information, and sharing such details without the patients’ permission is a breach of their autonomy. This type of conduct on the part of a health care team member is ethically unacceptable, even if the patient never learns about the circumstances, or no harm follows (4). To ensure accurate diagnosis and determine the appropriate course of treatment, patients should be able to confide the most intimate details to their physicians. Breach of patient confidentiality leads to non-presentation, which can bring about incorrect diagnoses and jeopardize all therapeutic measures (1).

It is also perceptible that confidentiality cannot be absolute, and breach of confidentiality may sometimes be morally or legally permissible. As a general rule, risk of a serious injury to the patient or others can justify a breach of confidentiality (4). The duties of physicians have been clarified in the World Medical Association International Code of Medical Ethics, and it is considered among the duties of a physician to respect patients’ secrets even after their death (6). 

Patients’ awareness of privacy and confidentiality can bring about a lot of advantages such as increased quality of healthcare services, decreased risk of irreversible physical and spiritual damages, promotion of patients’ health, and reduced hospital stay (7). Therefore, only patients who fully trust in physicians will reveal personal and intimate information regarding their health status. In addition, keeping patients’ matters confidential is crucial to maintain the faith of the public in medical professionals and healthcare services. An increasing number of studies have investigated the knowledge of patients regarding issues of privacy and confidentiality. Most of these studies have shown that patients have poor awareness of their rights (7 - 10), and some studies have indicated that many patients are either unaware of, or misunderstand the legal and ethical duty of conﬁdentiality. Furthermore, minority of patients distrust clinicians in terms of conﬁdential information protection and may consequently delay or forgo medical care (11). 

To our knowledge, most of the previous research on the Iranian population has investigated patients’ awareness of the Patients’ Rights Charter. Since no studies have specifically examined patients’ awareness of privacy and confidentiality, the present study was conducted to investigate these issues in an Iranian context. This study may provide an appropriate measure of patients’ awareness of their rights and the principles of medical ethics. An assessment of patients’ awareness can help policy makers devise appropriate regulations to improve physician-patient relationships. Moreover, the findings of this study may help finding necessary modifications for better respecting of patient privacy.

## Method

This was a cross-sectional study, and the study protocol was approved by the Ethics Committee of Tehran University of Medical Sciences. Since the level of patients’ awareness of their privacy and confidentiality had not been measured in previous studies, it was estimated to be 0.50. The sample size was calculated at 149, with a margin of error of 0.08, and confidence level of 95%. In order to enhance accuracy, the sample size was increased to 200, i.e., 100 patients in each hospital. The study population consisted of patients from the internal, surgical, and obstetrics and gynecology wards of Imam Khomeini and Shariati hospitals in Tehran during the year 2010. Patients were selected in a systematically random manner. The reasons for selecting these wards were the wide variety of patients and diseases, as well as the possibility of further violations of privacy and confidentiality.

Inclusion criteria consisted of patients' non-emergency admission, willingness to participate, and at least two days of hospitalization. 

Data were collected through a two-part questionnaire administered by two interviewers. The first part recorded demographic characteristics (age, gender, marital status, place of residence, and educational level), type of hospital ward, frequency of hospitalization, and duration of hospital stay. The second part consisted of ten multiple-choice questions. There were three options for response, scoring 1 for each correct answer, and 0 for each incorrect answer. To analyze data, patients were assigned to three categories of poor (0 ≤ scores ≤ 3), moderate (4 ≤ scores ≤ 7) and good (8 ≤ scores ≤ 10) levels of awareness. 

To determine the validity of the questionnaire, we used the content validity method. The content of the questionnaire was prepared on the basis of the research objectives through the study of textbooks and related articles, and evaluated by a panel of 5 experts in the field of medical ethics and epidemiology. The questionnaire was reviewed in terms of literary structure, clarity of the questions, and coverage of the items to be investigated. The final version constituted five questions on privacy and five on confidentiality. The section on privacy covered the definition, fields and violations of privacy, and women’s privacy issues. The part exploring confidentiality addressed patients’ awareness about respecting and observing confidentiality, disclosing patient's information and breaching confidentiality. Each question was designed to address one aspect of privacy or confidentiality, and the response options were devised in such a manner as to cover all dimensions of the issue (Tables 2 & 3).

A test - retest method was carried out to assess the external reliability of the questions in the ‘awareness’ part. Twenty patients with homogeneous characteristics were randomly selected in a systematic manner. They were asked to complete the questionnaire in two stages, seven to ten days apart. Pearson's correlation coefficient was used to evaluate the strength of concordance between the results of the two tests (> 0.7). The split-half method was used to determine the internal reliability. Subsequently, the reliability coefficient was determined at 0.723 and 0.724 respectively based on the Spearman-Brown formula and Cronbach's Coefficient Alpha. Thereafter, the trained interviewers completed the questionnaires; data were extracted and inputted into the database of SPSS version 21. To analyze data, we used descriptive statistics (mean, median, and frequencies), one-way ANOVA, Bonferroni, and chi-square test. 

In order to observe the principles of research ethics, all completed questionnaires were kept confidential and anonymous. In addition, at the beginning of the interview, the research scheme was presented, patients were asked to voluntarily cooperate in the study, and the interviewers attended the study hospitals after obtaining the required permissions. 

## Results

In this study, the mean age of the patients was 39.7 years. The largest group (29.2%) were 20 - 29 years old, and a few (6.7%) were aged less than 19. The demographic characteristics of the patients are presented in Table 1 below. 

**Table 1:** The demographic characteristics of the participants

		Frequency	
		N	%
Gender	Male	87	43.5
	Female	113	56.5
Marital Status	Single	59	29.5
	Married	135	67.5
	Uncertain	6	3.0
Place of Residence	Urban	179	89.5
	Rural	13	6.5
	Uncertain	8	4.0
Admission Ward	Internal	42	21.0
	Surgical	97	45.5
	Obstetrics and gynecology	55	27.5
	Uncertain	6	3.0
Frequency of Hospitalization	Once	143	71.5
	More than once	41	20.5
	Uncertain	16	8.0
Duration of Hospital Stay	< = 3 days	47	23.5
	4 - 5 days	67	33.5
	6 - 7 days	32	16.0
	> = 8 days	48	24.0
	Uncertain	6	3.0
Education Level	Illiterate	18	9.0
	Elementary school	22	11.0
	Middle school	26	13.0
	Junior high school	21	10.5
	Senior high school	59	29.5
	Associate degree	23	11.5
	Bachelor’s degree	26	13.0
	Master’s degree	3	1.5
	Uncertain	2	1.0

The overall response rate was 93.45% (200 out of 214). Table 2 shows the frequency of correct answers to each question in the ‘awareness’ part. One hundred fifty three patients (76.5%) provided a correct definition of privacy, and 161 patients (80.5%) were aware of instances of privacy violation (Table 3). In addition, 77 patients (38.5%) had good awareness of physician confidentiality. 

**Table 2:** The frequency of correct answers to each question in the questionnaire

**Field**	**Question Content**	**Correct**	
		**%**	N
Privacy	Awareness of privacy definition	153	76.5
	Awareness of privacy violations	161	80.5
	Awareness of privacy fields	76	38.0
	Awareness of privacy in medical settings	32	16.0
	Knowledge about women's privacy	81	40.5
Confidentiality	Awareness of physician confidentiality	77	38.5
	Awareness of patient's permission for breach of confidentiality by the physician	47	23.5
	Awareness of legal issues of confidentiality	28	14.0
	Awareness of confidentiality in specific cases	113	56.5
	Awareness of confidentiality violations	158	79.0

Also, only 46 patients (23.0%) believed that physicians could disclose patients' information to reduce or eliminate a significant risk of serious harm to others. Meanwhile, 47 patients (23.5%) did not think it was necessary for physicians to obtain patients’ consent before consulting with their families. Moreover, 105 patients (52.5%) did not believe that physicians needed patients’ permission to consult with their colleagues or other members of the medical team in cases of multidisciplinary diagnosis and treatment. 

Twenty-eight patients (14.0%) were aware that disclosing patient’s information is unethical, against religion, and illegal. One hundred and thirteen patients (56.5%) had previously known that medical information pertaining to mentally retarded patients should be recounted to their parents or guardians. Thirty-nine patients (19.5%) did not consider the results of medical examinations and tests as confidential in cases where patient security, employment, insurance issues and legal competency were concerned, and 47 patients (23.5%) were not aware that in research studies it is essential not to disclose patients’ identity (Table 3).

One hundred and fifty eight patients (79.0%) had good awareness of the confidentiality of examination results and medical consultations (except for the cases falling under the category of legal obligations). This study showed that 15 patients (7.5%) were not aware that in case of patients’ decision to commit suicide or homicide, physicians must inform the relevant authorities (Table 3).

**Table 3:** Sample questions about privacy and confidentiality included in the questionnaire

**#1**	**Questions and Options**
**1**	The definition of privacy is.... A. To stay away from the community and live alone B. The right to stand alone and practice control over personal information C. To hide all information from others
**2**	Which option is privacy violation? A. Interpretation of statements and actions of individuals B. Meddling in patients’ private affairs and disclosing their inconvenient information C. Obtaining information about the intellectual beliefs of individuals during medical examination
**9**	Which option is wrong about confidentiality? A. The health information of children and mentally retarded patients should not be disclosed to their parents or guardians. B. The results of medical examinations and tests may be disclosed to the requesting organization in situations pertaining to patient security, employment, insurance issues and legal competency. C. Patients’ individual profile should not be disclosed in medical research.
**10**	Which option is true about confidentiality? A. The physician should not disclose information about a patient who is considering suicide or homicide. B. The results of examinations and medical consultations are confidential, except in legal cases. C. Members of the medical staff who are not directly involved in the treatment process are allowed to access patient information.

*1 Number of question in the questionnaire*

Finally, in response to the question of whether male physicians should be allowed to perform physical examinations on female patients, 81 patients (40.5%) answered that they should, where it was a matter of saving lives. It may therefore be concluded that they had a good level of awareness in this regard.

The mean of the acquired score in the ‘awareness’ part was 4.61 ± 1.63 out of 10, and the median was 4. Table 4 demonstrates the frequency distribution of the patients’ total awareness.

**Table 4:** The frequency distribution of the patients’ total awareness

**Awareness**	**N**	**%**
**Poor**	42	21.0
**Moderate**	144	72.0
**Good**	14	7.0

No significant difference was found between the patients’ mean of awareness scores and variables including age, gender, marital status, place of residence, educational level, admission ward, frequency of hospitalization, and duration of hospital stay. 

The mean score of the postgraduate group (master’s degree) was the highest, and the greatest difference was observed between the two groups of illiterate and postgraduate patients (0.064).

## Discussion

In general, this study showed that patients had moderate awareness of privacy and confidentiality. It demonstrated that most of the patients provided a correct definition of privacy and confidentiality, but they were not aware of the more subtle instances of the two rights. 

Most of the previous research on Iranian patients has investigated their awareness of the Patients’ Rights Charter, and none has specifically dealt with their awareness of privacy and confidentiality. In the study by Parniyan et al. on 472 patients in two hospitals located in the city of Jahrom during 2014, the mean score of patients’ knowledge of their rights was 15.99 ± 5.82 (out of 24 scores) (12). Similarly, in a study by Mastaneh and Mouseli performed in two tertiary teaching hospitals affiliated with Shiraz University of Medical Sciences during 2012, 30.5% of the patients had weak, 59.4% moderate, and 10.1% good awareness of their rights (7). A study by Bateni et al. conducted on 385 patients in eight hospitals across the city of Isfahan during 2006 showed that over 50% of patients were not aware of the Patients' Rights Charter (13). Regarding patients’ awareness of the concepts of privacy and confidentiality, our findings are consistent with the above-mentioned studies. 

An Iranian study by Emami Razavi et al. performed on 70 medical assistants and 140 hospitalized patients from the emergency ward of Imam Khomeini Hospital investigated observance of some provisions of the Patients’ Rights Charter (including privacy and confidentiality). The results showed that 57.9% of the patients were not aware of their rights at all (14).

Findings of another Iranian study conducted by Mossadegh Rad et al. on 160 patients showed that 40% of them have very poor, 41.9% poor, and 18.1% moderate awareness of patients’ rights (15).

The findings of a study by Yaghobian et al. on 336 patients from four teaching hospitals of Sari in 2011 indicated that 58.9% of the patients had poor, 12% moderate, and 29.1% good knowledge about patients’ rights. The patients’ awareness about 'the right of privacy and confidentiality' and 'the right of confidentiality and the necessity of the patients’ permission for disclosure of information' were respectively 34.8% and 33.6% (16).

Another study conducted by Hojjatoleslami and Ghodsi on 416 inpatients in Hamadan Hospital indicated that 56.2% of the patients were not familiar with the Patients’ Rights charter, and only 29.3% were aware of it (17). Zeina et al. in South Egypt reported that 75.0% patients did not know about patients' rights (18). In Turkey, Kuzu et al. showed that few patients (9%) knew about their rights (19). The findings of these studies suggest that the level of the patients’ awareness of privacy and confidentiality was lower than that of the subjects in our study.

On the contrary, a 2012 study in Saudi Arabia conducted by Almoajel on 250 patients hospitalized in the country’s largest governmental hospital showed good awareness of confidentiality (80.3%) (20). Similarly, in Gonabad, Basiri Moghadam et al. showed in 2010 that only 5.6% of the patients had weak awareness, and most of the others had good awareness of the Patients’ Rights Charter (21). In Malaysia, a study by Yousuf et al. on 250 hospitalized patients showed that 90.8% patients were aware of their rights, and almost all of them appreciated privacy and confidentiality (22). The Ravaghi et al. study performed on 306 inpatients across three hospitals in Tehran indicated that the level of patients’ awareness of the right to privacy and confidentiality was 68.6% (23). In these studies, the level of patients’ awareness about their rights was higher than that of our study. These studies have not clarified the cause of the high level of patients' awareness of their rights, and it is unclear whether patients had received training regarding these issues. In these studies, the right to privacy and confidentiality has been part of the research, but our study focused on privacy and confidentiality issues, which may be the main reason for the difference between results. Although the patients in our study were generally familiar with privacy and confidentiality, they were little aware of the more subtle instances of these rights.

Zulfikar and Ulusoy believe that patients must first be aware of their own rights and responsibilities and then expect the physicians and nurses to observe them (24). According to our study, one-third of the patients did not assume the physician-patient relationship in the scope of privacy. Therefore, it is necessary to keep patients informed and educate them to better perceive these issues. 

In their study in Greece, Merakou et al. stated that 94.2% of the patients responded negatively to the question of whether they had ever claimed their rights. They also stated that in order to respect the rights of patients, it is essential to establish ethics committees, hire lawyers and experts who are aware of patients’ rights in hospitals, provide patients with information about their rights, and familiarize them with the relevant new rules (10).

In another study in the UK in which 30 patients were asked about confidentiality, all were in favor of physician confidentiality, and 83.0% demanded absolute confidentiality. However, in the case of child abuse, epileptic patients driving, drunk driving, etc., this percentage dropped to 69 (1).

In the United States, Fuzzell et al. showed that most patients concerned about their discussions to remain confidential and private (25). But our study showed that a little more than one third of the patients were aware of the right of privacy. 

Based on our findings, although patients knew that those not directly involved in the treatment process must not attend patients’ bedside, most patients did not raise any objections against this situation. 

In Australia, Knowles and McMahon found that the public is in favor of disclosing secrets in cases of child abuse or conspiracy, or when a patient confesses to murder, or intends to commit suicide or homicide (26). Furthermore, our study showed that patients knew that the relevant authorities must be informed of cases of homicide or suicide. In addition, patients agreed that the information of mentally retarded patients should be provided to their parents.

In consistence with the results of two review articles by Joolaee (8) and Abedi (9), our study showed that the level of patients' awareness about privacy and confidentiality is inadequate and has remained unchanged over recent years. 

## Conclusions

Based on the results and considering the patients’ weak or moderate awareness of privacy and confidentiality, it is the ethical and professional responsibility of the medical professionals to train and observe confidentiality and privacy issues, and thereby promote the observance of patients’ rights. In addition, it is necessary that both healthcare providers and recipients be informed about these issues. In terms of patients’ awareness, education may be provided upon admission or at any other appropriate time via provision of oral explanation as well as written media such as pamphlets, brochures, booklets, etc.

Health policy makers should develop and implement a plan for raising patients’ awareness of privacy and confidentiality to improve physician-patient relationships.

Finally, much remains to be learned about strategies to improve patient's awareness of their rights, and it is necessary to conduct further studies with a focus on the impact of interventions in this regard.
